# A prospective survey of comprehensive score for financial toxicity in Japanese cancer patients: report on a pilot study

**DOI:** 10.3332/ecancer.2018.847

**Published:** 2018-07-05

**Authors:** Kazunori Honda, Bishal Gyawali, Masashi Ando, Keiji Sugiyama, Seiichiro Mitani, Toshiki Masuishi, Yukiya Narita, Hiroya Taniguchi, Shigenori Kadowaki, Takashi Ura, Kei Muro

**Affiliations:** 1 Department of Clinical Oncology, Aichi Cancer Center Hospital, Nagoya, 464-8681, Japan; 2 Department of Medicine, Brigham and Women’s Hospital, Harvard Medical School, Boston, MA 02115, USA

**Keywords:** financial toxicity, cost, cancer, Japan, health insurance

## Abstract

**Background:**

Financial toxicity (FT) has a negative impact on the quality of life and survival of patients with cancer. The comprehensive score for FT (COST) questionnaire is a tool to measure FT which has already been validated in patients with cancer in the United States. However, the feasibility and validity of assessing FT using the COST questionnaire have not been established in non-US healthcare settings, including that in Japan.

**Methods:**

This is a prospective pilot survey to ascertain the feasibility of using the COST questionnaire to evaluate FT in Japanese patients with advanced solid cancer who had been receiving chemotherapy for at least 2 months. The COST questionnaire was translated into Japanese using Functional Assessment of Chronic Illness Therapy methodology.

**Results:**

Of the 12 patients approached, 11 (92%) responded to the questionnaire. The median COST score was 22 (range, 6–29; mean ± SD, 20.18 ± 8.17). Five (45%) and two (18%) patients suffered grade 1 (COST score 14–25) and grade 2 (COST score 1–13) FT, respectively. The COST measure demonstrated good internal consistency with a Cronbach *α* of 0.87.

**Conclusions:**

The COST measure demonstrated good feasibility in measuring FT in the Japanese healthcare setting. Despite the existing universal health insurance system and ceiling amount for high-cost medical expenses, some Japanese patients experienced meaningful FT during chemotherapy. A prospective study is already underway to confirm the preliminary results (UMIN: 000025043).

## Introduction

Financial toxicity (FT) is now a well-recognised problem in cancer care [1]. FT refers to the increased financial burden on cancer patients to cover the costs of treatment which adversely affects the patient’s lifestyle. However, FT includes not only objective financial burden but also a measure of the degree to which financial burden impacts patient well-being. Previously thought of as a problem of low-and-middle income countries where most patients cannot afford treatment, FT has now become a serious problem even among high-income countries mostly because of the increasing costs of newer cancer drugs, such as targeted agents or immunotherapies [2–4].

Studies in the USA have shown that many patients do not adhere to even life-saving oral cancer drugs such as imatinib due to increased drug prices [5]. Another study showed that 4,728 (2%) of 231,596 patients suffered bankruptcy and patients who suffered bankruptcy due to FT died earlier [6]. A study done in Italy, which has a universal health insurance system similar to that of Japan, has also shown that FT is associated with poor survival [7]. However, FT has not been well defined and unlike the Common Terminology Criteria for Adverse Events (CTCAEs) for physical toxicities, valid methods to quantify FT have not been well established. Hence, further studies into the methods to properly measure and quantify the risks of FT are important.

Japan has an excellent medical insurance system [8]. Under the Japanese universal healthcare system, all citizens and foreign residents are compulsorily required to join the national health insurance programme and they pay only 30% of the health care bill from their pocket. A patient older than 75 is required to pay only 10% of the health care bill from their pocket. In addition, Japan also has a unique system of ‘ceiling amount’ for high-cost medical expenses. This limit differs with the patient’s age and income; however, it ranges between 10,000 and 250,000 yen per month (approximately 100–250 USD). This ceiling amount is the maximum any patient has to pay from their pocket. That means, if a patient’s out-of-pocket medical care bill exceeds the ceiling amount, all other costs become free with public subsidies. However, these systems were established in 1973 before cancer treatment costs started to skyrocket and currently, there is a huge debate as to how long this remains a sustainable strategy for Japan. Despite these protections, we hypothesised that cancer patients in Japan are not immune from FT because the high price and improved efficacy of new cancer therapies mean that the patients must pay the ceiling amount every month for an increasing number of months. This could lead to serious financial burden for Japanese cancer patients. In addition, Japanese patients usually avoid discussion of cost for therapies for cultural reasons, thus FT has rarely been researched before.

The comprehensive score for FT (COST) score has been validated as a useful tool for measuring FT among patients with cancer in the USA [9, 10]. However, the health insurance system in the USA is unique and thus, COST score’s feasibility and validity in other countries with public universal health care in place is unknown. Thus, we aimed to evaluate the feasibility of the COST questionnaire as a tool to evaluate FT among patients with cancer in a different health care system (Japan) where a universal public health insurance system exists. Here, we report on the pilot phase of the study in which we assess the feasibility of using the Japanese version of the COST questionnaire in measuring FT among Japanese cancer patients.

## Methods

### Patients

Patients who were receiving ongoing chemotherapy in the Aichi Cancer Center Hospital were recruited. Eligibility criteria included patients receiving chemotherapy for at least 2 months and aged 20 or older. Patients receiving neoadjuvant and adjuvant chemotherapy were also eligible to participate. Patients in Japan are billed every time they visit the hospital or buy drugs from the pharmacy unlike in the USA where the bills arrive later, usually a few months after the treatment has been received. Thus, the financial impact on cancer patients in Japan can start from the very first visit and therefore, a time frame of at least 2 months is sufficient to at least get a preliminary idea of FT in Japanese cancer patients

### Procedure

Eligible patients provided informed consent and were handed the questionnaire which they could fill in at home and return by post. Postal envelopes with stamps already attached were provided to the patients from the institutional funds. No other funding support was received for this study. The study was approved by the Institutional Review Board of Aichi Cancer Center Hospital.

### COST questionnaire translation

We used the Functional Assessment of Chronic Illness Therapy methodology to translate the original COST questionnaire from English to Japanese by permission [11]. Two researchers (KH and MA) independently translated the original COST questionnaire in English to Japanese. Then, they integrated their translations. Next, backtranslation to English was performed which was reviewed by an English speaker (BG) for its similarity to the original. In addition, other co-authors reviewed the final Japanese version.

### Grading of FT

The COST score is 11 items patent-reported outcome measure to evaluate FT [9, 10]. Every item ranges from 0–4, thus the COST score can range from 0–44. A score of 0 represents the highest FT and a score of 44 represents the lowest FT. These scores have been subdivided into four grades, thus more than 26 means no impact on quality of life (Grade 0), 14–25 means mild impact (Grade 1), 1–13 means moderate impact (Grade 2) and 0 means high impact (Grade 3). According to this criterion, more than grade 1 FT was defined as positive FT.

### Data collection and analysis

Questionnaire items included COST score, out-of-pocket medical costs, total family income (six categories at 2 million yen intervals), total family savings (seven categories at 2 million yen intervals) (1 USD = 100 JPY approximately). Socioeconomic backgrounds were also collected and included marriage status, household size, educational status, work status and total assets. Information on the disease status was obtained from electronic medical charts and included primary site, local/metastatic, duration of chemotherapy and chemotherapy regimen at the time of the questionnaire.

### Statistical analysis

We defined more than 80% of survey response rate as the feasibility of using this questionnaire. The Japanese version of the COST score was assessed for internal consistency using the Cronbach *α*. Coefficients > 0.8 were regarded as good enough [12]. The Cronbach *α* was analysed by using R statistical software, ver. 3.3.2.

## Results

Patients were recruited between January and February 2017. Of the 12 patients approached, 11 (92%) responded to the questionnaire. Patients’ characteristics are listed in Tables 1 and 2. Median age was 65 (range 30–72) and 8 (73%) patients were female. Primary tumour sites were colorectal (*n* = 6: 55%), oesophageal (*n* = 2: 18%), thyroid (*n* = 2: 18%) and melanoma (*n* = 1: 9%). Eight patients (73%) received treatment with at least one molecular targeted agent, including bevacizumab, cetuximab and lenvatinib. The median length of time from the start of chemotherapy was 9 months (range, 2–55 months).

The median annual household income was between 4 and 6 million yen and median household saving was over 10 million yen. The median patient reported monthly cost of payment was 49,423 yen (range 24,694–375,000 yen, *n* = 10). The median COST score was 22 (range, 6–29; mean ± SD, 20.18 ± 8.17). Five (45%) and two (18%) patients suffered grade 1 and grade 2 FT, respectively. Four (36%) patients cut spending on food or clothing, four (36%) on leisure and seven (64%) used their savings to pay for cancer treatment. The COST measure demonstrated good internal consistency with a Cronbach *α* of 0.87.

## Discussion

This is the first report to evaluate FT using the COST questionnaire among patients with cancer in Japan where a universal public health insurance system exists. Our study showed that it was feasible to use the COST tool (translated into Japanese) to measure FT among Japanese cancer patients, with high response rate and internal consistency measured by Cronbach *α*. Although this was a pilot study with the primary objective of showing the feasibility of using the Japanese version of the COST tool, our preliminary data also showed that over 60% of patients reported more than grade 1 FT and used some strategies, such as cutting spending on food, clothing or leisure to cope with FT. Thus, a certain number of Japanese cancer patients might have been suffering from FT who have gone undetected and undiscussed.

It is proposed that financial burden due to treatment should be considered a ‘toxicity’ of treatment similar to physical adverse events [13]. Some authors have even proposed that FT should be reported along with physical toxicities in publications of clinical trials [14, 15]. However, although well-established tools such as CTCAE exist for reporting physical toxicities, there is no established tool for measuring FT in cancer patients. One method to measure FT is defined as health related spending making up more than 20% of the total family income [16]. Some other grading criteria for FT have also been proposed before [17] but none of them have been well-validated. However, FT does not involve only objectively measurable parameters. The range of health related expenses is ambiguous and FT also involves worsening of QoL and emotional upheavals due to financial burden. Fenn *et al* [18] asked cancer survivors simple four-degree questions about financial problems and discovered that 30% of patients reported some FT. In another study, Delgado-Guay *et al* [19] asked advanced cancer patients about their financial distress using a numeric scale (0–10) and 90% of patients reported some degree of FT. The reported frequency of FT varies between studies and the true frequency is unclear. Thus, for true assessment and intervention of FT, ‘we need more science and less art’ [20] and the first step to that end is the establishment and validation of a tool to capture FT with acceptable accuracy.

The COST tool was developed by de Souza *et al* [9, 10] in order to objectively quantify FT in cancer patients and they have now already validated it among advanced cancer patients in the USA. Huntington *et al* [21] applied this COST tool to measure FT in 100 multiple myeloma patients [21]. The COST tool successfully evaluated FT and was correlated with the use of strategies to cope with treatment expenses, such as borrowing money. However, it was unknown whether the same tool could be used in other countries that have a different health care and medical insurance system. In previous reports of the COST questionnaire from the USA, the median COST scores were 23.5 and 23 [10, 21]. The COST score in our pilot study was 22 which is similar to the reports from the USA. The COST measure demonstrated good internal consistency with a Cronbach *α* of 0.87. The Japanese version of the COST questionnaire showed good reliability and was justified to continue further research.

Measuring FT will shed light onto undetected and undiscussed problems in cancer patients in Japan where the financial implications of cancer treatment are rarely discussed. The COST questionnaire can be used as a screening tool for FT, and patients with FT would be referred to social caregivers to discuss the appropriate use of public services to address financial issues. Furthermore, knowing what factor has the most influence on FT (such as age or income) can play a key role in shaping policies to address FT.

According to recent reports, average monthly income levels for an elderly Japanese couple over the age of 60 is about 180,000 yen, mainly from pensions. Average monthly consumption expenditure is about 240,000 yen, thus leading to an average monthly deficit of 60,000 yen. Elderly Japanese people probably spend their savings to compensate for this deficit, and even 10% out of pocket costs could impact the quality of their daily lives. According to a recent report from the Japanese Ministry of Internal Affairs and Communications, 13.2% of elderly Japanese patients pay 10% of their medical bill out of their own pocket. On the other hand, the average monthly income level for an elderly Japanese couple who are not retired is about 500,000 yen and the average monthly consumption expenditure is about 340,000 yen, thus leading to a monthly surplus of 160,000 yen. Japanese health care systems protect patients, especially the elderly; from out-of-pocket expenditures for healthcare well; however, a continuous burden can induce FT, especially among the retired population. Furthermore, FT does not only arise from out-of-pocket expenditures for healthcare. It can be induced from conditions such as loss of job or anxiety about the future.

An important limitation of our study is the small sample size because of the nature of our study being a pilot survey. We also did not seek to retest the internal consistency of the COST tool because the 11 questions included in the COST tool seemed relevant to our setting as well; our objective was to perform an external validation of the tool in the Japanese healthcare context. Another important limitation of our study is that it was a single centre survey and the results may not be generalisable to the whole of Japan. However, based on this pilot study, we have launched a prospective validation study to validate these results in a larger sample (UMIN: 000025043). In the future, we also plan to expand this study across Japan to produce reliable evidence that can be implemented nationwide. If the relevance of the COST questionnaire can be proven in a country with a public health insurance system like Japan, we could use this tool as a common metric to discuss FT across countries with a private insurance model like the USA and a public insurance model like Japan or some European countries. Also, just by chance, most of our patients were female.

## Conclusion

In conclusion, our Japanese translation of the COST tool demonstrated good feasibility in measuring FT in Japanese patients with cancer. We thus show that the Japanese translation of the COST tool can be used to conduct research on FT in Japan in the future. Our preliminary data also showed that despite the existing universal healthcare system and ceiling amount for high-cost medical expense, some Japanese patients may experience meaningful FT during cancer drug therapy. This is a preliminary report with a small number of patients; a prospective study is already underway to confirm the preliminary results

## Conflicts of interest

The authors have no conflicts of interest to disclose for this study.

## Funding

This study was supported by internal funds from the Department of Medical Oncology, Aichi Cancer Center, Japan.

## Figures and Tables

**Table 1.** Patients’ characteristics.

Total	*N* = 11
Age (years, range)	65(30–72)
Sex	
Male	3
Female	8
Marital status	
Married	6
Nonmarried	5
Household income per year (yen)	
<2,000,000 (about 20,000 dollars)	1
2,000,000–3,999,999 (about 20,000–39,999 dollars)	3
4,000,000–5,999,999 (about 40,000–59,999 dollars)	4
6,000,000–7,999,999 (about 60,000–79,999 dollars)	2
8,000,000–9,999,999 (about 80,000–99,999 dollars)	0
≧10,000,000 (about 100,000 dollars)	0
Not reported	1
Employment status	
Employed full-time	1
Employed part-time	0
Retired due to cancer	4
Retired due to not cancer	3
Not working outside home	1
Not reported	1
Education	
Junior high school	1
High school	3
College or junior college	3
University or graduate school	4
Primary site	
Colorectal	6
Oesophageal	2
Thyroid	2
Melanoma	1
Time to first chemotherapy (months)	9(2–55)
Chemotherapy including molecular target agent	
Yes	8
No	3

**Table 2.** Patients’ characteristics and COST score.

Patient number	COST score	Age	Sex	Primary site	Duration of chemotherapy (months)	Chemotherapy regimen at survey	Cost of drugs per one cycle (10 thousand yen)	Interval of chemotherapy (weeks)
1	15	41	F	colorectal	5	S-1, oxaliplatin, bevacizumab	35	3
2	6	54	M	oesophageal	9	docetaxel	7	3
3	28	67	F	colorectal	8	Fluorouracil, leucovorin, bevacizumab	19	2
4	27	66	F	melanoma	2	Dacarbazine	5	4
5	22	72	F	colorectal	29	Capecitabine, oxaliplatin, bevacizumab	36	3
6	24	47	M	thoyroid	6	Lenvatinib	63	4
7	8	30	F	colorectal	24	S-1, oxaliplatin, bevacizumab	35	3
8	29	65	M	oesophageal	55	Fluorouracil, nedaplatin	7	4
9	14	55	F	colorectal	7	Fluorouracil, leucovorin, Oxaliplatin, irinotecan, cetuximab	32	2
10	27	65	F	thoyroid	9	Lenvatinib	63	4
11	22	67	F	colorectal	9	Trifluridine-tipiracil, irinotecan, bevacizumab (clinial trial)	28	2

## References

[1] Zafar SY (2015). Financial toxicity of cancer care: it’s time to intervene. J Natl Cancer Inst.

[2] Gyawali B (2017). Low-value practices in oncology contributing to financial toxicity. Ecancermedicalscience.

[3] Prasad V, De Jesus K, Mailankody S (2017). The high price of anticancer drugs: origins, implications, barriers, solutions. Nat Rev Clin Oncol.

[4] Gyawali B, Sullivan R (2017). Economics of cancer medicines: for whose benefit?. New Bioeth.

[5] Dusetzina SB, Winn AN, Abel GA (2014). Cost sharing and adherence to tyrosine kinase inhibitors for patients with chronic myeloid leukemia. J Clin Oncol.

[6] Ramsey SD, Bansal A, Fedorenko CR (2016). Financial insolvency as a risk factor for early mortality among patients with cancer. J Clin Oncol.

[7] Perrone F, Jommi C, Di Maio M (2016). The association of financial difficulties with clinical outcomes in cancer patients: secondary analysis of 16 academic prospective clinical trials conducted in Italy. Ann Oncol.

[8] Ikegami N, Yoo BK, Hashimoto H (2011). Japanese universal health coverage: evolution, achievements, and challenges. Lancet.

[9] de Souza JA, Yap BJ, Hlubocky FJ (2014). The development of a financial toxicity patient-reported outcome in cancer: the COST measure. Cancer.

[10] de Souza JA, Yap BJ, Wroblewski K (2017). Measuring financial toxicity as a clinically relevant patient-reported outcome: The validation of the Comprehensive Score for financial Toxicity (COST). Cancer.

[11] Eremenco SL, Cella D, Arnold BJ (2005). A comprehensive method for the translation and cross-cultural validation of health status questionnaires. Eval Health Prof.

[12] Tavakol M, Dennick R (2011). Making sense of Cronbach’s alpha. Int J Med Educ.

[13] Zafar SY, Peppercorn JM, Schrag D (2013). The financial toxicity of cancer treatment: a pilot study assessing out-of-pocket expenses and the insured cancer patient’s experience. Oncologist.

[14] Gyawali B J Glob Oncol.

[15] Saltz LB (2016). Perspectives on cost and value in cancer care. JAMA Oncol.

[16] Banthin JS, Bernard DM (2006). Changes in financial burdens for health care: national estimates for the population younger than 65 years, 1996 to 2003. JAMA.

[17] Khera N (2014). Reporting and grading financial toxicity. J Clin Oncol.

[18] Fenn KM, Evans SB, McCorkle R (2014). Impact of financial burden of cancer on survivors’ quality of life. J Oncol Pract.

[19] Delgado-Guay M, Ferrer J, Rieber AG (2015). Financial distress and its associations with physical and emotional symptoms and quality of life among advanced cancer patients. Oncologist.

[20] de Souza JA, Yap B, Ratain MJ (2015). User beware: we need more science and less art when measuring financial toxicity in oncology. J Clin Oncol.

[21] Huntington SF, Weiss BM, Vogl DT (2015). Financial toxicity in insured patients with multiple myeloma: a cross-sectional pilot study. Lancet Haematol.

